# Effect of the Elastic Deformation of a Point-Sharp Indenter on Nanoindentation Behavior

**DOI:** 10.3390/ma10030270

**Published:** 2017-03-07

**Authors:** Takashi Akatsu, Shingo Numata, Yutaka Shinoda, Fumihiro Wakai

**Affiliations:** 1 Faculty of Art and Regional Design, Saga University, 1 Honjo-machi, Saga 840-8502, Japan; 2 Laboratory for Materials and Structures, Institute of Innovative Research, Tokyo Institute of Technology, R3-24 4259 Nagatsuta, Midori, Yokohama 226-8503, Japan; numata.s.zz@m.titech.ac.jp (S.N.); shinoda.y.ac@m.titech.ac.jp (Y.S.); wakai.f.aa@m.titech.ac.jp (F.W.)

**Keywords:** nanoindentaion, elastic deformation, finite element method, numerical analysis

## Abstract

The effect of the elastic deformation of a point-sharp indenter on the relationship between the indentation load *P* and penetration depth *h* (*P*-*h* curve) is examined through the numerical analysis of conical indentations simulated with the finite element method. The elastic deformation appears as a decrease in the inclined face angle *β*, which is determined as a function of the elastic modulus of the indenter, the parabolic coefficient of the *P*-*h* loading curve and relative residual depth, regardless of *h*. This indicates that nominal indentations made using an elastic indenter are physically equivalent to indentations made using a rigid indenter with the decreased *β*. The *P*-*h* curves for a rigid indenter with the decreased *β* can be estimated from the nominal *P*-*h* curves obtained with an elastic indenter by using a procedure proposed in this study. The elastic modulus, yield stress, and indentation hardness can be correctly derived from the estimated *P*-*h* curves.

## 1. Introduction

Nanoindentation is a form of mechanical testing characterized as a depth-sensing indentation [1] to evaluate local mechanical properties through the analysis of the indentation load *P* versus the penetration depth *h* (*P*-*h* curve, hereafter). The analysis is principally based on a geometrical definition in which the indentation is carried out on a flat surface using an indenter geometrically defined such as flat-ended, spherical, ellipsoidal, point-sharp (e.g., conical, Berkovich, Vickers, cube corner, etc.). The point-sharp indentation has an advantage in local mechanical testing owes to the analytical simplicity for the geometrical similarity [2].

**Figure A1:**
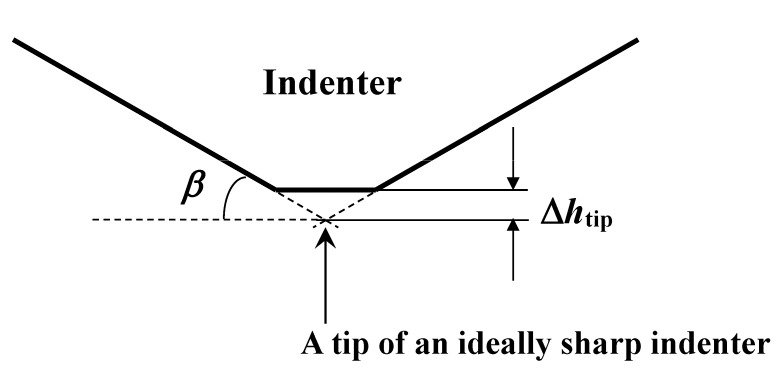
Schematic illustration of a truncated conical indenter tip.

**Figure A2:**
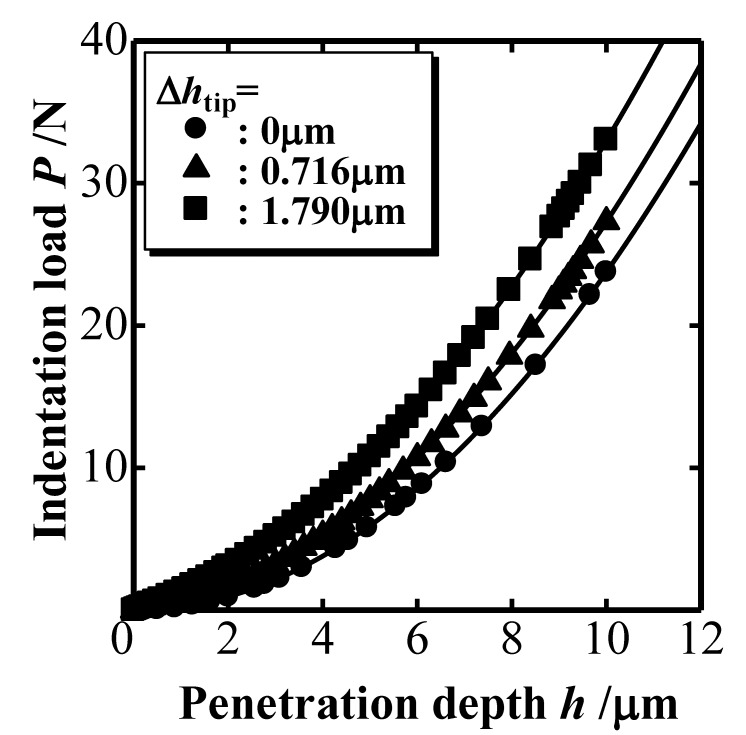
Indentation load *P* vs. penetration depth *h* curves obtained with truncated conical indenters.

**Figure A3:**
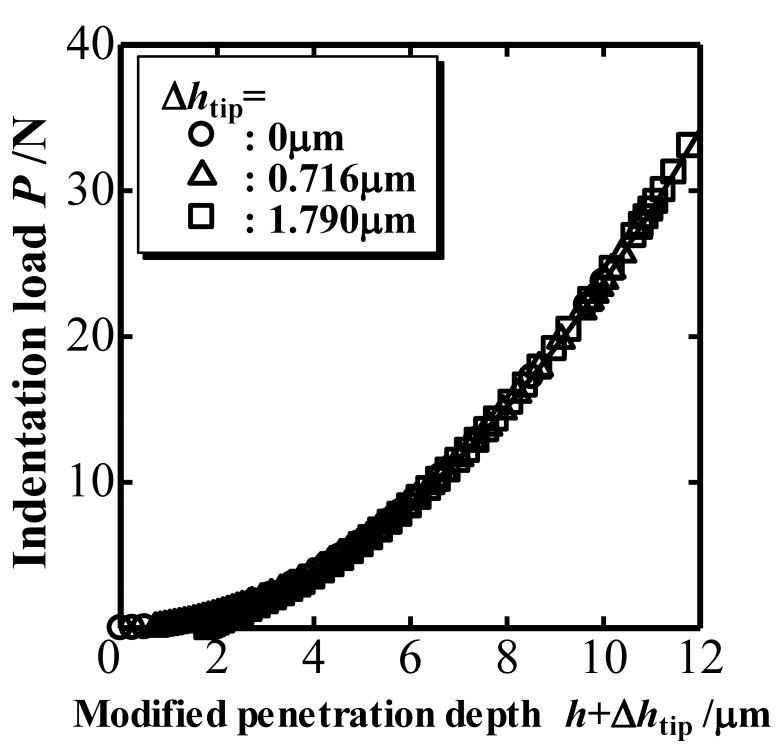
Indentation load *P* vs. modified penetration depth *h* + Δ*h*_tip_ curves obtained with truncated conical indenters.

**Figure A4:**
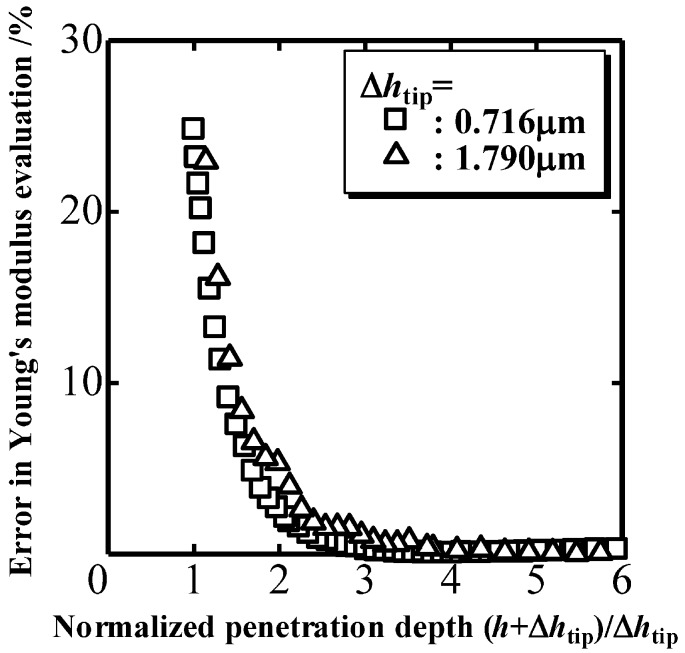
Error in Young’s modulus evaluation with a truncated conical indenter as a function of normalized penetration depth (*h* + Δ*h*_tip_)/Δ*h*_tip_.

**Figure A5:**
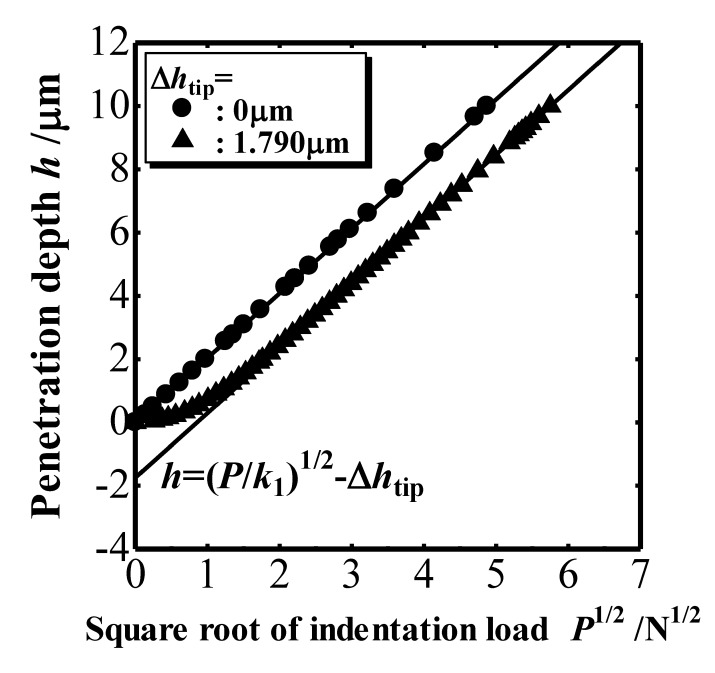
Penetration depth *h* vs. square root of indentation load P*** obtained with truncated indenters.

The bluntness of the indenter tip is one of the inevitable problems of undesirable tip geometry, especially for the point-sharp indentations, because it is impossible to make an ideally sharp indenter. The degree of the bluntness of a point-sharp indenter has been expressed in terms of the radius of curvature at the tip [3,4,5], but the actual geometry of a blunt tip is not guaranteed to be spherical. An area function [6,7] which gives the projected contact area at the maximum indentation load is another approach to express the bluntness of a point-sharp indenter, but the area function is theoretically valid only for hardness evaluation. A truncated tip which represents a blunt tip in an extremely poor situation [8] is a suitable model for a strict discussion on the effect of the tip bluntness on indentation behavior. According to the appendix of this paper, where a truncated tip is considered, the undesirable effect of the bluntness of a point-sharp indenter can be removed out simply if the *P*-*h* curve is shifted with Δ*h*_tip_ in the *h* direction for indentations deeper than 2Δ*h*_tip_, where Δ*h*_tip_ is the distance between ideally sharp and blunt tips (see Figure A1, Figure A2, Figure A3 and Figure A4). In addition, Δ*h*_tip_ can be estimated through an extrapolation of the linear relationship between *h* and P*** observed in the large *P* and *h* region to *P* = 0 (see Figure A5).

The elastic deformation of an actual point-sharp indenter, which has been conventionally taken into account on the basis of Hertzian contact [6]; Hertzian contact was basically used for spherical indentations as a modification of the elastic modulus evaluation. It is also an inevitable problem of undesirable tip geometry, especially for indentations on a very hard material, and there is still some controversy whether the modification based on the Hertzian contact can be applied to point-sharp indentations. Moreover, there are no reports on the modifications of the indenter elastic deformation for other mechanical properties such as the indentation hardness or yield stress. The geometrical changes of a point-sharp indenter due to elastic deformation should be considered when evaluating local mechanical properties with the nanoindentation technique.

In this paper, the effect of the elastic deformation of a point-sharp indenter on a *P*-*h* curve is quantified in a numerical analysis of conical nanoindentation behaviors simulated with the finite element method (FEM). In addition, a procedure of deriving physically meaningful *P*-*h* curves, which should be utilized for mechanical property evaluation. Finally, the validity and accuracy of this method is examined.

## 2. FEM Simulation of Nanoindentation

A conical indentation on a cylindrical elastoplastic solid was modeled in order to avoid the difficulty of modeling a pyramidal indenter widely used for actual nanoindentations. The FEM simulation exploited the large strain elastoplastic capability of ABAQUS code (Version 5.8.1) in the same way as reported in the literature [9,10]. Indentation contact was simulated by the use of elastic cone indenters with two different inclined face angles *β* (19.7° and 30°). Young’s modulus of the elastic indenter was in the range of 300–1140 GPa. The finite-element mesh in the elastic indenter with *β* of 19.7° was composed of 775 4-node quadrilateral axisymmetric elements with 2443 nodes. The elastic indenter with *β* of 30° had 704 elements with 2258 nodes.

The FEM simulation used elastoplastic linear strain hardening rules, i.e., σ=Eε for σ<Y, and $\sigma =Y+E_{p}\varepsilon_{p}$ for σ≥Y, where *σ* is the stress, *E* the Young’s modulus and *ε* the strain. Here, *Y* is the yield stress and *E*_p_ ($\equiv d\sigma /d\varepsilon_{p}$) is the plastic strain hardening modulus, where *dσ*, *dε*, *dε*_e_, and *dε*_p_ are, respectively, the incremental values of stress, total, elastic, and plastic strains. Indentations were simulated for *E*, *Y* and *E*_p_ ranges of 50–1000 GPa, 0.1–60 GPa, and 0–200 GPa, respectively. The von Mises criterion with isotropic hardening was used to determine the onset of yielding flow.

## 3. Results and Discussion

A quadratic relationship between *P* and *h* on loading is theoretically guaranteed for a point-sharp indentation on the flat surface of a homogeneous elastoplastic solid [11,12]. The quadratic relationship was also observed in simulated *P*-*h* curves made with an elastic cone indenter. This indicates that the elastic deformation of a cone indenter can be described as a decrease in *β* determined regardless of *h*. Therefore, nominal indentations made with an elastic cone indenter with an original inclined face angle *β*_o_ should be physically equivalent to indentations made with a rigid cone indenter with the decreased inclined face angle *β*_d_.

**Figure 1:**
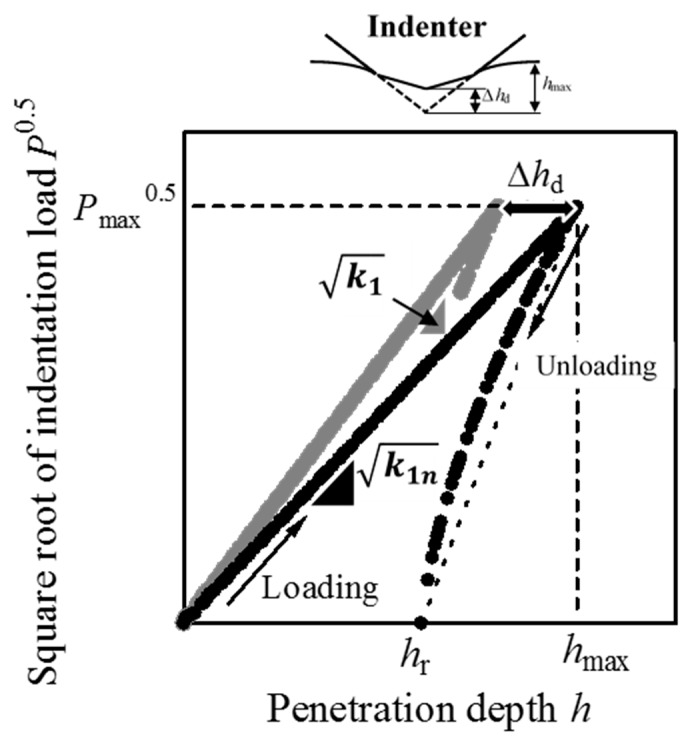
Schematic illustration of the effect of indenter elastic deformation on a *P*-*h* curve.

A nominal quadratic *P*-*h* relationship for an elastic cone indenter can be depicted as follows:

(1) $$P=k_{1n}h^{2}\text{ for loading},$$

where *k*_1n_ is the nominal indentation loading parameter. Here, *h* in Equation (1) is the nominal penetration depth because the decrease in *β* from *β*_o_ to *β*_d_, due to the elastic deformation of a cone indenter, gives a decrease in real penetration depth. Thus, a physically meaningful *P*-*h* relationship can be written with a true indentation loading parameter *k*_1_, which should be observed in a *P*-*h* loading curve using a rigid cone indenter with *β*_d_ as

(2) $$P_{\max}=k_{1}{\left(h_{\max}-\Delta h_{d}\right)}^{2},$$

where Δ*h*_d_ is the decrease in *h* at the maximum penetration depth *h*_max_ due to the elastic deformation of a cone indenter (see Figure 1). The combination of Equations (1) and (2) leads to the equation:

(3) $$k_{1}=k_{1n}{\left(1-\frac{\Delta h_{d}}{h_{\max}}\right)}^{-2}.$$

This means that Δ*h*_d_/*h*_max_ is a key parameter to estimating *k*_1_ from the nominal *k*_1n_. In other words, Δ*h*_d_/*h*_max_ can be simulated as

(3′) $$\frac{\Delta h_{d}}{h_{\max}}=1-\sqrt{\frac{k_{1n}}{k_{1}}},$$

where *k*_1n_ in Equation (3’) is observed in a simulated *P*-*h* loading curve with an elastic cone indenter and *k*_1_ in Equation (3’) is evaluated with the mechanical properties inputted into the FEM model [9,10]. In the following paragraph, the effect of Δ*h*_d_/*h*_max_ on a *P*-*h* curve is examined quantitatively through numerical analysis.

In addition to *k*_1_, the relative residual penetration depth *ξ*, defined as *h*_r_/*h*_max_, where *h*_r_ is the residual penetration depth, characterizes a *P*-*h* curve and nominally decreased by the elastic deformation of a cone indenter to be *ξ*_n_. A true *ξ*-value, which should be observed in a *P*-*h* curve using a rigid cone indenter with *β*_d_, can also be evaluated with the mechanical properties inputted into the FEM model [9,10]. The numerical analysis revealed that the evaluated *ξ* can be correlated with the nominal *ξ*_n_ as a function of Δ*h*_d_/*h*_max_

(4) $$\xi =\xi_{n}{\left\{1-{\left(\frac{\Delta h_{d}}{h_{\max}}\right)}^{0.85}\right\}}^{-0.50}.$$

**Figure 2:**
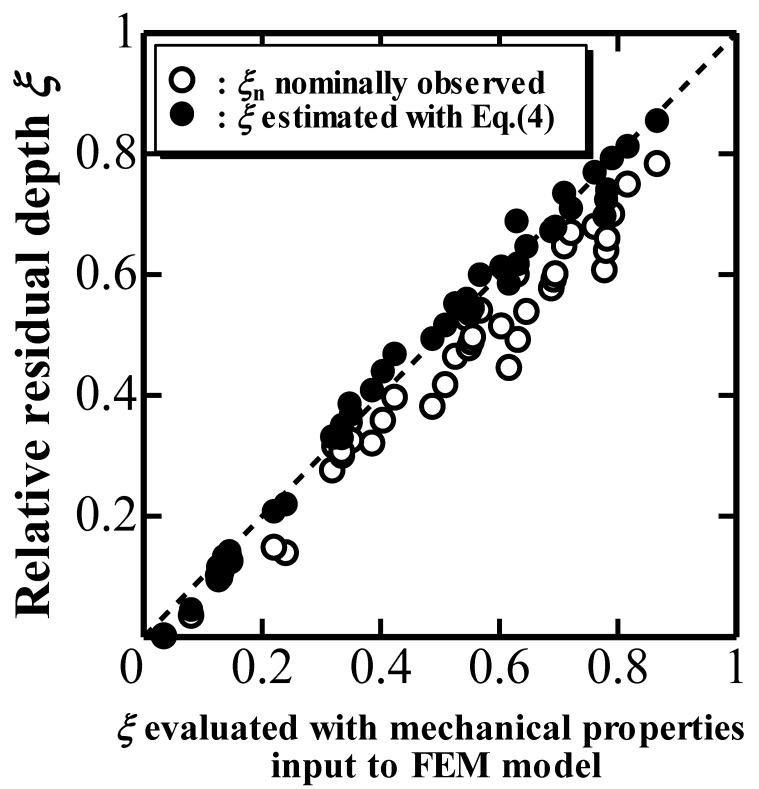
Relative residual depth *ξ* estimated with Equation (4) and *ξ*_n_ nominally observed plotted against *ξ* evaluated with mechanical properties inputted into the FEM model.

Figure 2 plots *ξ* estimated with Equation (4) and *ξ*_n_ against the true *ξ* evaluated with the mechanical properties inputted into the FEM model [9,10]. The results indicate the validity of using Equation (4) to estimate *ξ* from the nominal *ξ*_n_ and Δ*h*_d_/*h*_max_. In addition, it is confirmed that *ξ*_n_ is smaller than *ξ* because of the overestimation of the penetration depth *h* due to the elastic deformation of the indenter. Moreover, a true indentation unloading parameter *k*_2_ defined as *P*_max_/(*h*_max_ − *h*_r_)*, which should be observed in an *P*-*h* unloading curve using a rigid cone indenter with *β*_d_, can be estimated from a simulated *P*-*h* curve with an elastic cone indenter characterized by *k*_1n_ and *ξ*_n_ using Equations (3) and (4) as

(5) $$k_{2}=\frac{k_{1}}{{\left(1-\xi \right)}^{2}}=k_{1n}{\left(1-\frac{\Delta h_{d}}{h_{\max}}\right)}^{-2}{\left[1-\xi_{n}{\left\{1-{\left(\frac{\Delta h_{d}}{h_{\max}}\right)}^{0.85}\right\}}^{-0.50}\right]}^{-2}.$$

**Figure 3:**
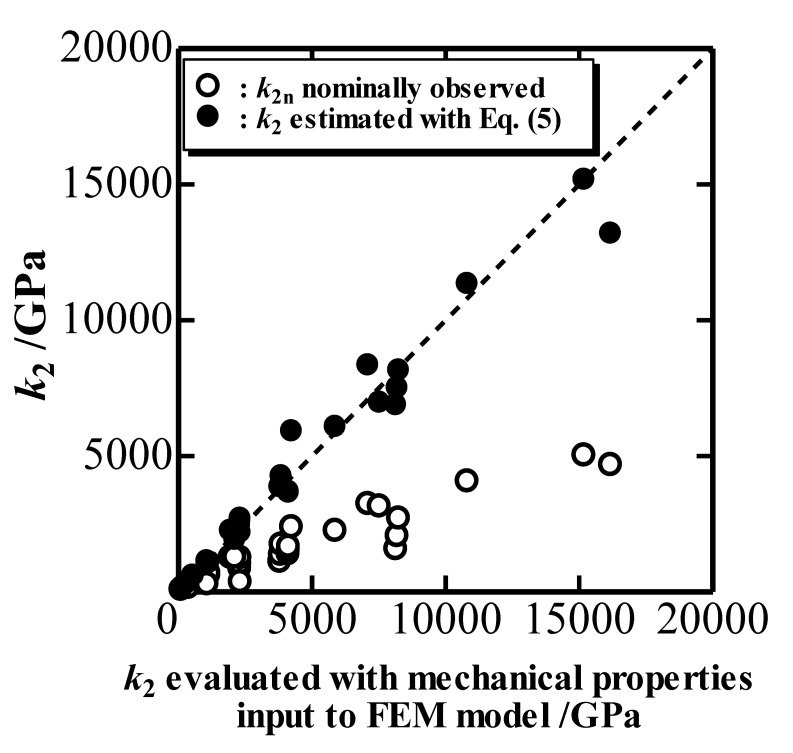
Indentation unloading parameter *k*_2_ simulated and *k*_2n_ nominally observed plotted against *k*_2_ evaluated with mechanical properties inputted into the FEM model.

Figure 3 plots the estimated *k*_2_ with Equation (5) as well as the nominal indentation unloading parameter *k*_2n_ determined from a simulated *P*-*h* curve with an elastic cone indenter against the true *k*_2_ evaluated with the mechanical properties inputted into the FEM model [9,10]. Figure 3 indicates that *k*_2_ can be estimated correctly by using Equation (5) with Δ*h*_d_/*h*_max_, and that the nominal *k*_2n_ is quite far from *k*_2_ owing to the overestimation of *h*.

**Figure 4:**
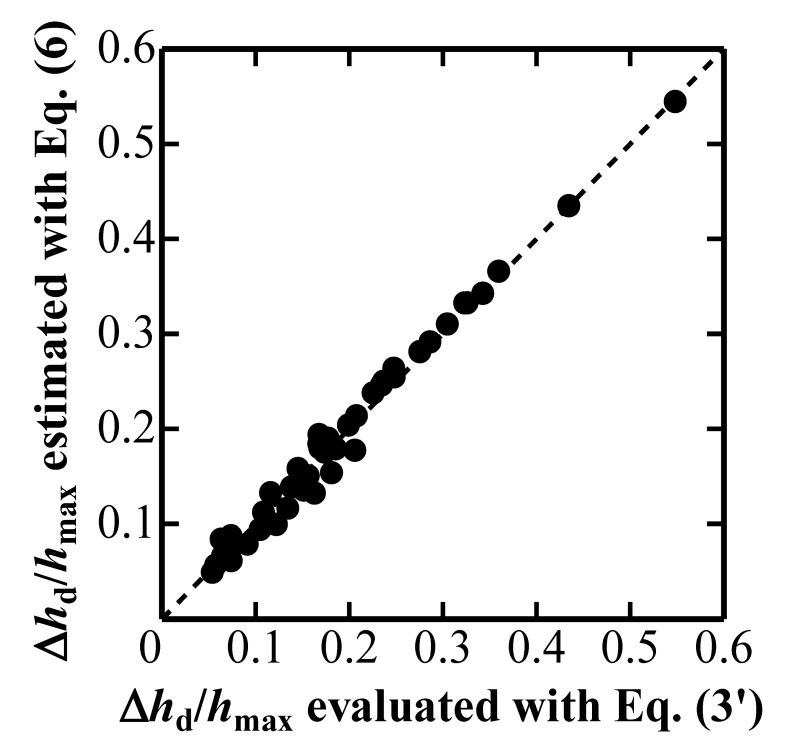
Degree of elastic deformation of conical indenter Δ*h*_d_/*h*_max_ estimated with Equation (6) plotted against Δ*h*_d_/*h*_max_ evaluated with Equation (3’).

The numerical analysis also revealed that Δ*h*_d_/*h*_max_ is determined to be

(6) $$\frac{\Delta h_{d}}{h_{\max}}=0.616{\left\{\frac{\;k_{1n}}{{E_{i}}^{\prime }\left(1+{\xi_{n}}^{1.5}\right)}\right\}}^{0.84},$$

where *E*_i_′ is defined as *E*_i_/(1 − *ν*_i_*) and *E*_i_ and *ν*_i_ are Young’s modulus and Poisson’s ratio of an elastic indenter, respectively. Figure 4 plots Δ*h*_d_/*h*_max_ estimated with Equation (6) against Δ*h*_d_/*h*_max_ evaluated with Equation (3’). Figure 3 indicates that Δ*h*_d_/*h*_max_ can be estimated by using Equation (6) with a nominally observed *P*-*h* curve characterized by *k*_1n_ and *ξ*_n_, and with the elastic properties of an elastic indenter characterized by *E*_i_ and *ν*_i_.

In order to estimate mechanical properties from a *P*-*h* curve characterized with *k*_1_, *k*_2_ and *ξ*, we should know the inclined face angle *β*_d_ of the elastically deformed indenter. Numerical analysis revealed that *β*_d_ is given as a function of *β*_o_, *ξ*_n_ and Δ*h*_d_/*h*_max_

(7) $$\frac{\tan\beta_{d}}{\tan\beta_{o}}=1-{\left(1-{\xi_{n}}^{0.80}\right)}^{0.83}{\left(\frac{\Delta h_{d}}{h_{\max}}\right)}^{0.90}.$$

**Figure 5:**
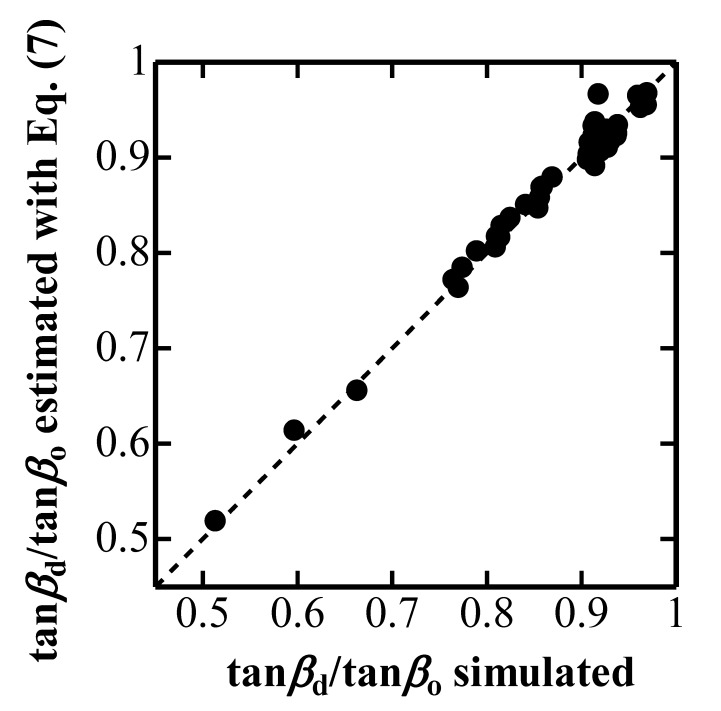
Degree of change in inclined face angle tan*β*_d_/tan*β*_o_ estimated with Equation (7) plotted against tan*β*_d_/tan*β*_o_ observed in simulated nanoindentation.

Figure 5 plots $\frac{\tan\beta_{d}}{\tan\beta_{o}}$ estimated with Equation (7) against $\frac{\tan\beta_{d}}{\tan\beta_{o}}$ observed in a simulated nanoindentation, and indicates the validity to estimate the inclined face angle *β*_d_ of the elastically deformed indenter with Δ*h*_d_/*h*_max_.

**Figure 6:**
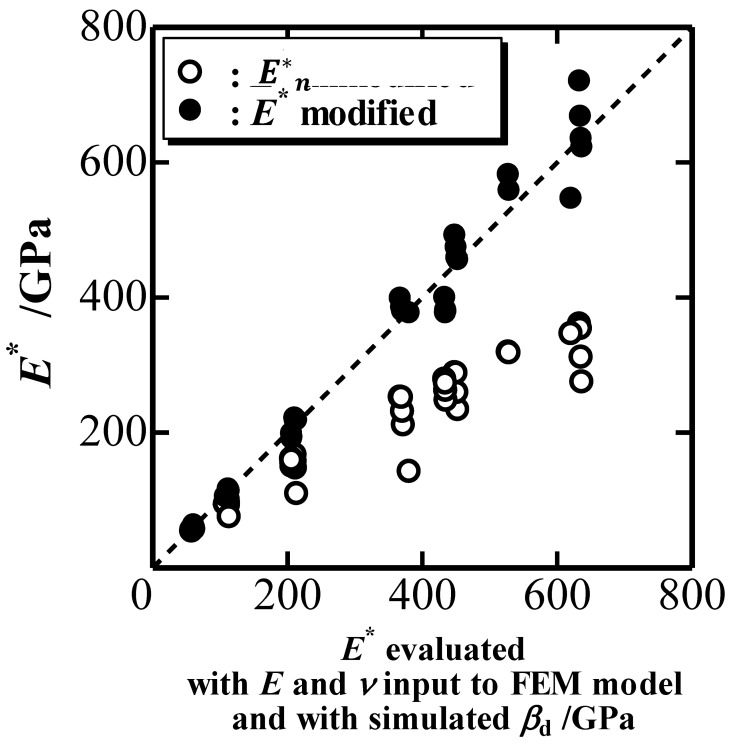
Representative indentation elastic modulus E* estimated with simulated *P*-*h* curves plotted against E* evaluated with mechanical properties inputted into the FEM model.

The representative indentation elastic modulus E*, defined as $E^{*}=\frac{E}{1-{\left(\nu -0.225\tan^{1.05}\beta_{d}\right)}^{2}}$ in terms of *β*_d_ [9], can be estimated from the simulated *P*-*h* curve using Equations (3)–(7) if we know *E*_i_ and *ν*_i_, whereas it is evaluated with *E* and *ν* inputted into the FEM model and with *β*_d_ observed in simulated nanoindentations. Figure 6 plots the estimated E* (black circles) against the evaluated E*. The white circles are E*_n_ estimated with the nominal values of *k*_2n_ and *ξ*_n_ [9], which means that the elastic deformation of an indenter is not modified for the estimation of E*. Figure 6 indicates the modification of the elastic deformation of an indenter can determine E* correctly. The underestimation of E* without the modification (the white circles in Figure 6) is caused by the overestimation of the elastic rebound during the unloading process because the extrinsic elastic deformation of the indenter is added to the intrinsic elastic deformation of the indented material.

**Figure 7:**
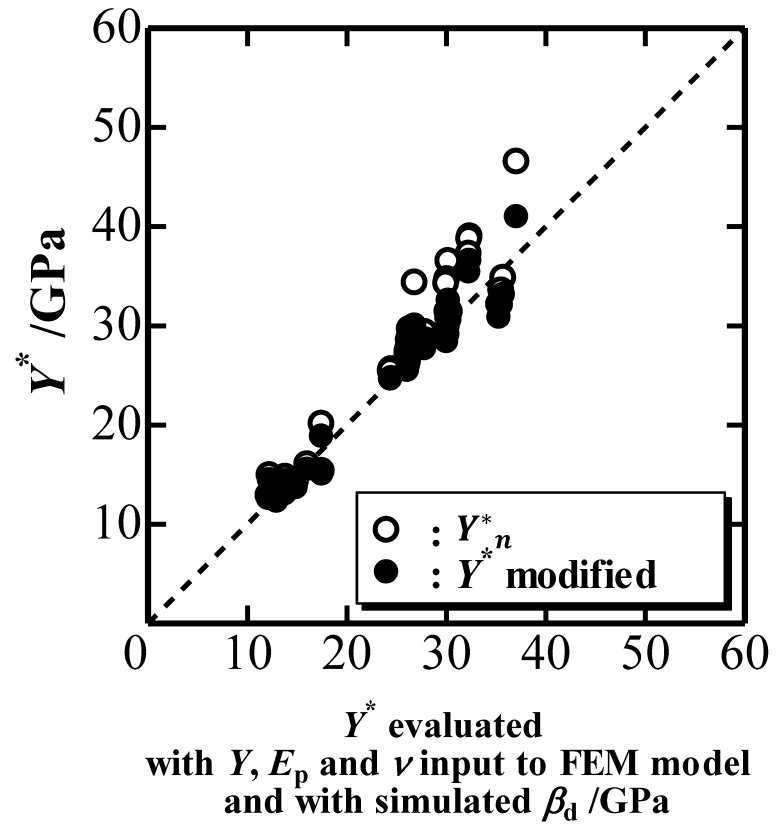
Representative indentation yield stress Y* estimated with simulated *P*-*h* curves plotted against Y* evaluated with mechanical properties inputted into the FEM model.

The representative indentation yield stress Y*, defined as $Y^{*}=\frac{Y+0.25E_{p}\tan\beta_{d}}{1-\left(\nu -0.225\tan^{1.05}\beta_{d}\right)}$ in terms of *β*_d_ [10], can also be estimated using a simulated *P*-*h* curve and Equations (3)–(7) if we know *E*_i_ and *ν*_i_. Moreover, it can be evaluated with *Y*, *E*_p_ and *ν* inputted into the FEM model and with *β*_d_ of the simulated indentation. Figure 7 plots the estimated Y* (black circles) against the evaluated Y*. Y*_n_ estimated with E*_n_, *ξ*_n_ and *β*_o_ is plotted for comparison. This figure shows that the modification of the elastic deformation of an indenter more or less correctly estimates Y* although the difference between the modified and unmodified Y* is not so large with respect to the difference observed in E* (see Figure 6). A relatively large difference in Y* is typically found in the range of *ξ* less than 0.1, where plastic deformation is not dominant. The small difference observed in Figure 7 is attributed to the decrease in *k*_1n_ and *ξ*_n_ due to elastic deformation of an elastic indenter, where the former decreases Y* nominally while the latter increases Y* apparently.

**Figure 8:**
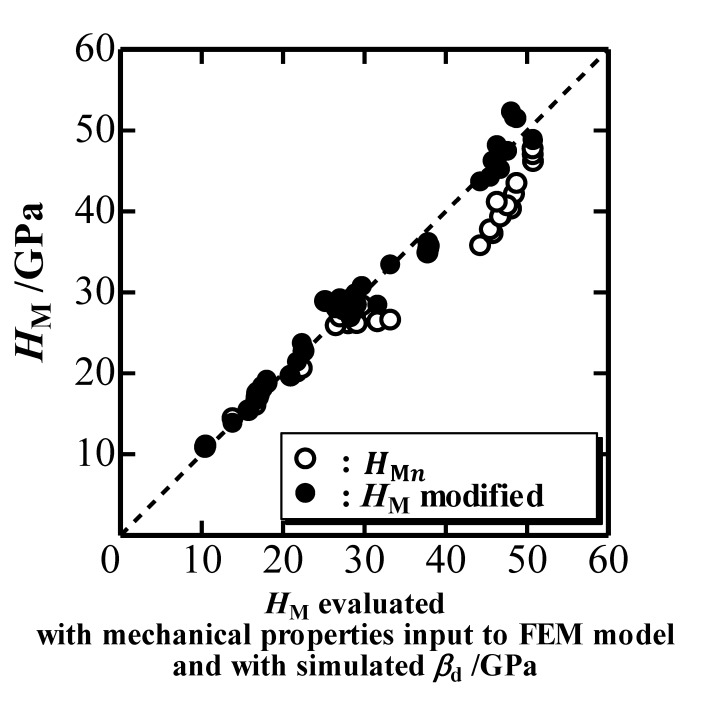
Indentation hardness *H*_M_ estimated with simulated *P*-*h* curves plotted against *H*_M_ evaluated with mechanical properties inputted into the FEM model and with simulated *β*_d_.

A previous study on the indentation hardness *H*_M_ found that it can be evaluated with the mechanical properties inputted into the FEM model and with the simulated *β*_d_ [9,10]. On the other hand, *H*_M_ can be estimated from a true *P*-*h* curve characterized with *k*_1_, *k*_2_ and *ξ*. Figure 8 plots the estimated *H*_M_ (black circles) against the evaluated *H*_M_. The nominal *H*_Mn_ estimated with the nominal *P*-*h* curve is plotted as white circles in Figure 8, and a comparison reveals that the modification more or less correctly estimates *H*_M_, although the difference between the estimated *H*_M_ and the nominal *H*_Mn_ is not so large with respect to the difference observed in E* (see Figure 6). The difference is rather high in the large *H*_M_ region, where elastic deformation of the indenter is most severe. The small difference observed in Figure 8 owes to the decrease in *k*_1n_ and *ξ*_n_ due to elastic deformation of an elastic indenter, where the former decreases *H*_M_ nominally while the latter increases *H*_M_ apparently through the decrease of nominal contact depth.

**Figure 9:**
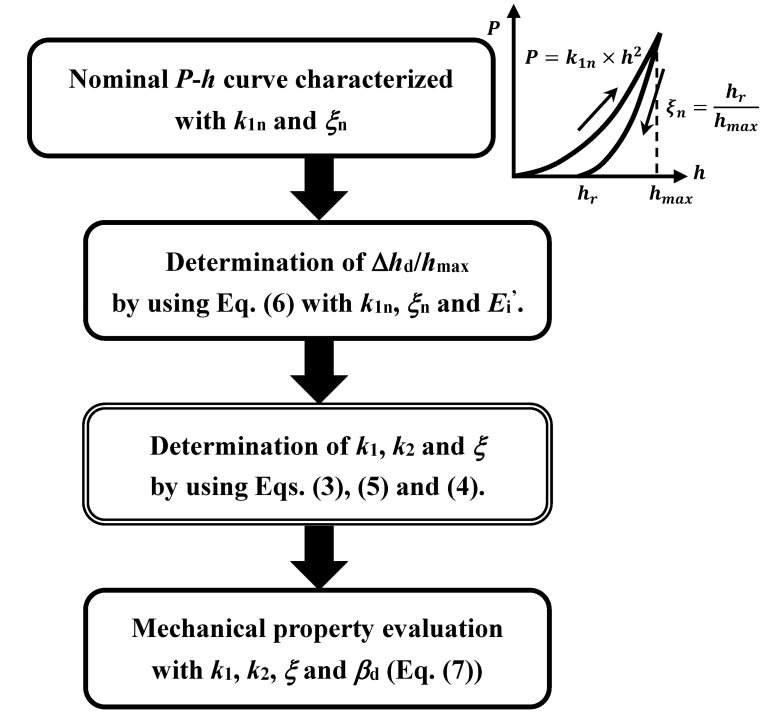
Flow chart of the procedure to evaluate mechanical properties.

We conducted nanoindentation experiments and reported E*, Y* and *H*_M_ for several materials [9,10]. These values were evaluated with modified *P*-*h* curves (see Figure 9) considering elastic deformation of a diamond indenter with Young’s modulus and a Poisson’s ratio of 1140 GPa and 0.07, respectively. Table 1 shows these mechanical properties as well as those evaluated with a nominal *P*-*h* curve made without considering any elastic deformation of the indenter. Δ*h*_d_/*h*_max_ and *β*_d_ estimated with the numerical analysis developed in this study are shown in order to examine the degree of the elastic deformation of the indenter. Δ*h*_d_/*h*_max_ and the change in *β* (Δ*β* = *β*_o_ − *β*_d_) are large for relatively hard materials (e.g., fused silica and alumina), which would cause a large elastic deformation of the indenter. Even in that case, the changes in Y* and *H*_M_ due to the elastic deformation of the indenter are not so large. In contrast, the change in E* is so large that it cannot be ignored. The underestimation of E* without the modification is caused by the overestimation of the elastic deformation during the unloading process because the extrinsic elastic deformation of the indenter is added to the intrinsic elastic deformation of the indented material.

According to Equation (6), the following equation can be derived

(8) HMEi′=γ2g(1+ξn1.5)  (Δhd0.616hmax)10.84.

When indentation hardness is not affected much by the indenter elastic deformation, where *γ* is the surface profile parameter defined as *γ* = *h*_max_/*h*_c_, *h*_c_ is the contact depth, and *g* is the geometrical factor of a point-sharp indenter to be 24.5 for *β* = 19.7°. *E*_i_’ is required to be about 250 times larger than *H*_M_ for Δ*h*_d_/*h*_max_ smaller than 0.05, where the effect of the indenter elastic deformation on a *P*-*h* curve may be ignored for indentations with Berkovich-type indenter.

## 4. Conclusions

The effect of the geometrical changes due to the elastic deformation of a point-sharp indenter was examined by conducting a numerical analysis of *P*-*h* curves simulated with FEM. The effect appears as a decrease in the inclined face angle *β*. The key parameter Δ*h*_d_/*h*_max_, which can be utilized to derive the physically meaningful *P*-*h* curve and the decreased *β*, can be estimated with an equation derived by numerical analysis. The mechanical properties of indented materials, such as E*, Y* and *H*_M_, can be estimated by using the *P*-*h* curve and *β* characterized by *k*_1_, *k*_2_ and *ξ* estimated with the key parameter Δ*h*_d_/*h*_max_. The modification of a *P*-*h* curve and *β* with Δ*h*_d_/*h*_max_ is most effective for the estimation of the accurate E* with respect to Y* and *H*_M_.

## Figures and Tables

**Table 1:** Effect of elastic deformation of diamond indenter on mechanical property evaluations.

Materials	Δ*h*_d_/*h*_max_	*β*_d_/*β*_o_ (deg.)	*k*_2_/*k*_2n_ (10 ^3^ GPa)	*ξ*/*ξ*_n_	E* (GPa)	Y* (MPa)	*H*_M_ (GPa)
					(Modified Value/Unmodified Value)		
Brass	0.020	19.6/19.7	7.65/4.78	0.930/0.913	102/81	584/597	1.26/1.29
Duralumin	0.020	19.6/19.7	4.37/3.03	0.909/0.893	77/64	605/618	1.31/1.33
Beryllium copper alloy	0.027	19.6/19.7	9.49/5.44	0.925/0.904	136/103	841/863	1.82/1.86
Fused silica	0.065	19.0/19.7	0.615/0.477	0.550/0.522	74/65	5.21 × 10^3^/5.35 × 10^3^	8.56/8.40
Alumina	0.158	18.3/19.7	4.90/2.25	0.690/0.614	340/225	12.6 × 10^3^/12.9 × 10^3^	24.3/22.8

## Appendix A. Effect of the Tip Bluntness of a Point-Sharp Indenter on Nanoindentation Behavior

An indentation on a linearly elastic solid with a Young’s modulus *E* and Poisson’s ratio *ν* of 100 GPa and 0.499, respectively, was simulated using a rigid indenter with a truncated tip [8] in order to represent a tip in an extremely poor situation. The simulation was basically carried out in the same way described in the Section 2 of this paper. The inclined face angle *β* of the indenter was set to be 19.7°, which is the Vickers–Berkovich equivalent angle. The distance Δ*h*_tip_ between ideally sharp and truncated tips (see Figure A1) was set to be 716 or 1790 nm with respect to the maximum penetration depth of 10 μm.

The *P*-*h* curves shown in Figure A2 were obtained by simulating an indentation on a perfectly elastic body with a Poisson’s ratio *ν* of ≈0.5 for a series of Δ*h*_tip_. We only examined a perfectly elastic body because the effect of bluntness would be most severe in this case. As shown in Figure A3, the curve deviates from that for an ideally sharp indenter (Δ*h*_tip_ = 0) as Δ*h*_tip_ increases and the amount of the shift in the *h* direction almost coincides with Δ*h*_tip_. The results in this figure indicate the effect of a blunt tip of a conical indenter on the *P*-*h* curve can be simply described as a change in the penetration depth with Δ*h*_tip_, especially in the large *P* and *h* region. The error in Young’s modulus is evaluated as $\frac{\left|E_{P-h}-E_{\mathrm{inp}}\right|}{E_{\mathrm{inp}}}$ (expressed as a percentage), where *E_P_*_-*h*_ and *E*_inp_ are Young’s modulus values obtained from the simulated *P*-*h* curve and inputted into an FEM model, respectively. Figure A4 plots this error as a function of penetration depth normalized by Δ*h*_tip_. It indicates that a reliable Young’ modulus can be obtained with an error less than 10% from the *P*-*h* curve in the *h* region larger than 2Δ*h*_tip_. In other words, *P*-*h* data for *h*-values shallower than 2Δ*h*_tip_ should be omitted in order to obtain reliable mechanical properties. It may be possible to get more reliable *P*-*h* data in the shallow *h* region for other geometries, but the *P*-*h* data are actually obscure and depend on the unknown geometry of the indenter tip. We simulated a truncated tip of a conical indenter because it represents the worst case of a blunt point-sharp indenter; consequently, the *P*-*h* data obtained in the *h* region larger than 2Δ*h*_tip_ guarantees accuracy in all cases. Figure A5, where *h* is plotted as a function of the square root of *P*, is an extrapolation of the linear relationship between *h* and *P**** observed in the large *P* and *h* region to *P* = 0. It gives Δ*h*_tip_ as an absolute value on the *h*-axis and is a way to estimate Δ*h*_tip_ from the *P*-*h* curve. We have already reported an example of estimating Δ*h*_tip_ from an experimental *P*-*h* curve [13]; Δ*h*_tip_ is estimated to be about 50 nm for the Berkovich indenter that we actually used for the nanoindentation experiment.
